# Ecological analysis of mosquito larval communities in Burkina Faso to inform environmental monitoring of genetic control programs

**DOI:** 10.1038/s41598-026-35602-6

**Published:** 2026-01-13

**Authors:** Inoussa Toé, Mahamadi Kientega, Armel Judicael Lingani, Jeremy Bouyer, Nouhoun Traoré, Aboubacar Karabinta, Abdoul Azize Millogo, Jacques Kaboré, Abdoulaye Diabaté, Hamidou Maiga

**Affiliations:** 1 Institut de Recherche en Sciences de la Santé, Bobo-Dioulasso, Burkina Faso; 2 Université Nazi Boni, Bobo-Dioulasso, Burkina Faso; 3 Université Joseph Ki-Zerbo, Ouagadougou, Burkina Faso; 4 Centre International de Recherche Développement sur l’Elevage en Zone Subhumide, Bobo-Dioulasso, Burkina Faso; 5 ASTRE, Cirad, INRAE, University of Montpellier, Plateforme Technologique CYROI, Sainte-31 Clotilde, La Réunion, France; 6 Institut des Sciences des Sociétés, Ouagadougou, Burkina Faso

**Keywords:** *Anopheles coluzzii*, Gene drive, Non-target organism, Ecological exposure score, Environmental monitoring, Ecology, Ecology, Zoology

## Abstract

In Burkina Faso, the development of the gene drive technology targeting *Anopheles coluzzii* raises important ecological questions about potential non-target effects. Understanding interactions in mosquitoes’ natural environments is crucial for developing effective post-release environmental safety monitoring. This study assesses the ecological exposure and potential risks to non-target organisms associated with *An. coluzzii* suppression. Using co-occurrence, niche overlap metrics, and characterisation of physicochemical parameters, we evaluated interspecific relationships among mosquitoes and macroinvertebrate taxa from larval habitats in Burkina Faso. Combined index revealed distinct ecological relationships, ranging from competitive or facilitative coexistence to spatial segregation driven by predation or behavioural avoidance. Based on these interactions, an exposure score was developed to quantify the potential susceptibility of non-target organisms to ecological changes following the removal of *An. coluzzii*. The results showed variable exposure among taxa, with *An. gambiae s.s.* having the highest score, followed by *An. arabiensis* and *Culex* spp. Predatory taxa such as Corixidae showed niche overlap but limited spatial co-occurrence, suggesting effective predation. The detection of hybrid forms (*An. coluzzii* x *An. gambiae s.s.*) further highlights the potential for gene flow. This study introduces a quantitative framework that combines ecological indices and exposure scores to predict potential risks to non-target organisms.

**Supplementary Information:**

The online version contains supplementary material available at 10.1038/s41598-026-35602-6.

## Introduction

Mosquito bites are responsible for most vector-borne diseases. Africa is one of the most severely affected regions, with high burdens and mortality rates over the years^1,2^. Mosquitoes are widely recognised as important vectors of human disease, transmitting pathogens that cause diseases, such as malaria, dengue fever, and lymphatic filariasis^3^. In sub-Saharan Africa, *Anopheles gambiae s.l.* plays a key role in spreading malaria, which remains a significant public health challenge^4^. Nevertheless, *Anopheles* species can breed in the same locations as other mosquito genera, such as *Culex* and *Aedes*. These species also serve as important vectors for diseases, including arboviruses and lymphatic filariasis.

In natural breeding habitats, competition among mosquito larvae is affected by resource availability (water, food, and shelter), water quality, and species-specific ecological adaptations. Understanding the ecological characteristics of mosquito larval habitats, such as electrical conductivity, dissolved oxygen levels, pH, salinity, CO_2_, total dissolved solids, turbidity, and temperature, is essential for developing effective vector control strategies^5,6^. These dynamics significantly influence larval growth, survival rates, and population patterns, ultimately affecting the emergence of adult mosquitoes, species composition, fitness, and the broader transmission of vector-borne diseases^7,8^.

Co-occurrence refers to two or more species living or appearing together in the same place or habitat, at the same time. Deviations from the independent occurrence of two species may indicate an ecological relationship such as predation, symbiosis, or competition. Studying co-occurrence patterns can thus provide valuable insights into the ecological connections among mosquito species and with their predators, especially when their responses to environmental factors are studied in parallel, helping to identify key factors influencing species distribution^9^. The co-occurrence approach has been used to investigate potential species interactions within an ecological system. A notable co-occurrence can indicate positive interactions (mutualism and facilitation), while competition or predation are regarded as negative interactions^10^.

Distribution patterns observed within a single ecosystem can be understood as species’ responses to environmental conditions or to dispersal limitations. The co-occurrence foundation suggests that when species within a community interact in ways that impact each other’s abundance or presence across various spaces, thereby shaping local community assembly patterns, their co-occurrence will not be random^11^.

This phenomenon can be identified through appropriate sampling designs and statistical analyses^11^. For example, it may be observed that predators are found alongside their prey more frequently than expected. At the same time, competitors are generally seen together less often than would be expected from a random assembly^12^. The results of the study by Freilich et al., highlight that a supplementary approach might complement the co-occurrence analysis, as some factors could influence the observed interactions identified as environmental variables, such as abiotic parameters, including pH, temperature, conductivity, turbidity, salinity, and % oxygen^12^.

Recent advances in vector control methods, particularly gene drive technology, provide a promising strategy for reducing disease transmission by suppressing or replacing mosquito populations^13–15^. In Burkina Faso, the development of gene drive technology specifically focuses on *An. coluzzii*, the predominant vector in the region^16,17^. However, implementing gene drive strategies within the target species could disrupt the existing ecological balance by altering competitive or predatory interactions with other mosquitoes and macroinvertebrates^18–20^. This raises a question: “Could the targeted suppression of *An. coluzzii* cause an ecological imbalance, promote the emergence of new vectors, or disrupt local aquatic ecosystems?” Indeed, such changes might enable other mosquito species to emerge and occupy the ecological niches left vacant by the decline in the target species, a phenomenon known as competitive release^21–23^. Such unforeseen shifts in mosquito dynamics could influence disease transmission patterns and jeopardise broader ecosystem stability, including predators that may be harmed by their suppression. Several genetic control initiatives are progressing towards field evaluations. The PRONTI (Priority Ranking of Non-target Invertebrates) system has been proposed as a framework for monitoring these interventions. Initially developed to prioritise non-target invertebrates for biosafety testing of transgenic crops^24^ and later adapted for classical biological control programmes^25^. PRONTI offers a structured approach for ranking organisms based on their risk, ecological or economic importance, and suitability for monitoring. It helps users follow a systematic process to assess and balance these factors within the framework of proposed post-release monitoring programmes.

This study investigates species competition, niche overlap, and co-occurrence patterns within the *An. gambiae s.l*. species and among other mosquitoes and macroinvertebrates in natural larval habitats. Additionally, we examined some physicochemical parameters of the larval habitats and their influence on species distribution and larval competition. We used the results to better characterise two PRONTI components for the collected species, the hazard, that is, the degree to which the suppression of *An. coluzzi* poses a threat to non-target organisms, and the exposure, meaning how much non-target organisms may be exposed to the stressor. This research contributed to a deeper understanding of mosquito ecology and provided essential information for post-release environmental safety monitoring of genetic tools for mosquito vector control in Burkina Faso.

## Results

### Ecology of the larval habitats

Several types of ecological niches were sampled during the survey for larval collection. These sites included seven types of mosquito larval habitats (*N* = 138), especially the artificial habitats (*N* = 13), ponds (*N* = 14), puddles (*N* = 70), rice paddy fields (*N* = 3), river pockets (*N* = 9), streams (*N* = 11), and tyre tracks (*N* = 18). Most of these sites were human-made, except for the streams and river pockets. Analyses of the ecological properties of these breeding sites revealed differential ecological patterns that may influence the abundance of the different macroinvertebrate communities. Table 1 presents the average values of each measured parameter at various breeding sites, including temperature, turbidity, pH, conductivity, and oxygen level. The average temperature ranged from 26.04 ± 2.56 °C to 33.82 ± 3.01 °C across the different types of breeding sites. The highest temperature was observed in the river/pocket, while the lowest was in ponds. Turbidity levels varied, with an average maximum of 362.95 ± 312.18 NTU, observed in the streams. Mean pH, conductivity, and % oxygen ranged from 7.46 ± 0.50 to 8.23 ± 0.53, 61.77 ± 14.97 to 271.17 ± 246.93, and 10.37 ± 5.63 to 16.96 ± 13.94, respectively. The highest pH and conductivity of 8.23 ± 0.53 and 271.17 ± 246.93, respectively, were observed in the river pockets. The temperature was shown to influence mosquito dynamics, with significant correlations observed between temperature and *Anopheles* spp. density. (*r* = 0.212, *p* = 0.012) and *Culex* spp. (*r* = 0.187, *p* = 0.028). *Anopheles coluzzii* exhibited a significant negative correlation with pH (*r* = − 0.223, *p* = 0.009), while a strong positive correlation was observed with turbidity (*r* = 0.344, *p* < 0.001) and temperature (*r* = 0.213, *p* = 0.012). In contrast, for *An. arabiensis*, a significant correlation with conductivity was detected (*r* = 0.267, *p* = 0.002). The complete Spearman correlation data, including coefficients and *p*-values, are provided in Supplementary file S1.

**Table 1:** Average and standard deviation of physicochemical parameters across habitat types from July to October 2024.

		pH^a^	Temperature (°C)^a^	Conductivity (µS/cm)^a^	Turbidity (NTU)^a^	Oxygen (%)^a^
Larval habitats *N* = 138	Artificial habitats *N* = 13	7.75 ± 0.53	28.55 ± 3.74	83.30 ± 87.38	197.73 ± 243.51	16.96 ± 13.94
	Ponds *N* = 14	8.20 ± 1.31	26.04 ± 2.56	64.52 ± 64.24	305.20 ± 378.78	21.11 ± 38.96
	Puddles *N* = 70	7.70 ± 0.59	31.13 ± 4.25	98.81 ± 231.83	285.72 ± 294.04	16.69 ± 14.89
	Rice paddy field *N* = 3	7.58 ± 0.38	31.07 ± 1.46	61.77 ± 14.97	184.40 ± 153.18	13.93 ± 2.90
	Streams *N* = 11	7.58 ± 0.63	31.36 ± 4.50	90.60 ± 208.30	362.95 ± 312.18	12.34 ± 5.73
	Tyre tracks *N* = 18	7.46 ± 0.50	29.88 ± 4.25	63.81 ± 90.93	274.98 ± 238.17	10.37 ± 5.63
	River/pocket *N* = 9	8.23 ± 0.53	33.82 ± 3.01	271.17 ± 246.93	32.20 ± 15.58	16.61 ± 3.52
	*p*-value^b^	0.082	< **0.001**	0.053	**0.017**	0.068

^a^Mean±Standard deviation^b^ Kruskal–Wallis rank sum test N = Number of le larval habitats. Bold values indicate statistically significant results (p < 0.05).

### Species diversity and distribution

**Fig. 1:**
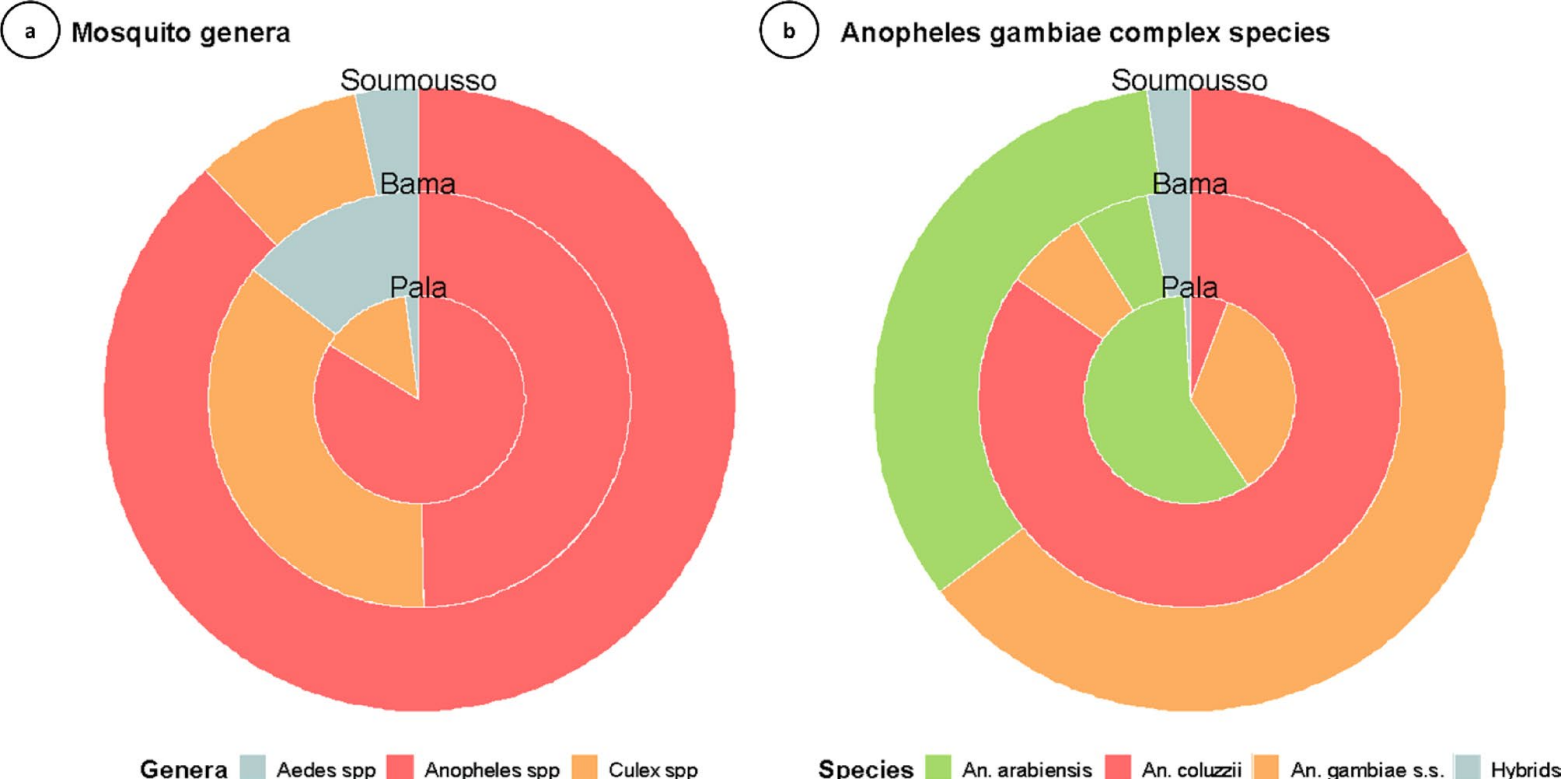
Distribution of mosquitoes by sampling site according to two taxonomic levels: mosquito genera and *Anopheles gambiae* complex species. Nested donuts show a circle representing different sites, each from inside to outside (Pala → Bama → Soumousso).

A total of 7,748 larvae from three mosquito genera were collected in Pala, Bama, and Soumousso. Figure 1 depicts the distribution of mosquito genera and species from these locations. Significant differences were observed between sites, indicating substantial variation in the composition of genera and species across the sampling areas. *Anopheles* spp. was the most common mosquito genus (Kruskal-Wallis chi-squared = 188.79, df = 2, *p*-value < 2.2e−16), representing 70.41% (5,455/7,748) of the recovered larvae, followed by *Culex* spp. and *Aedes* spp. mosquitoes, which made up 21.77% (1,687/7,748) and 7.82% (606/7,748), respectively. *Anopheles* mosquitoes were predominant at all sites, accounting for 50% (1671/3368) in Bama, 84% (1481/1768) in Pala, and 88.2% (2303/2612) in Soumousso. A subsample of *Anopheles* larvae (*n* = 1306 larvae) was subjected to molecular analyses for the identification of *An. gambiae* complex species.

Among these, *An. coluzzii* was the most prevalent species (Kruskal-Wallis chi-squared = 73.406, df = 3, *p*-value = 7.96e-16), comprising 41.03% (537/1,309), while *An. gambiae s.s.* and *An. arabiensis*, accounted for 29.79% (390/1,309) and 26.81% (351/1,309), respectively. *Anopheles gambiae s.s.* and *An. arabiensis* were predominant in Soumousso, with frequencies of 47% (287/607) and 33% (203/607), respectively. Conversely, in Bama, however, *An. coluzzii* was predominant, constituting 84.71% (421/497), and in Pala, *An. arabiensis* was the most common at 58.5% (120/205), followed by *An. gambiae s.s.* at 34.6% (71/205). A small proportion of hybrids between *An. coluzzii* and *An. gambiae s.s*. was detected, accounting for 2.37% (31/1,309) of the mosquitoes analysed.

**Fig. 2:**
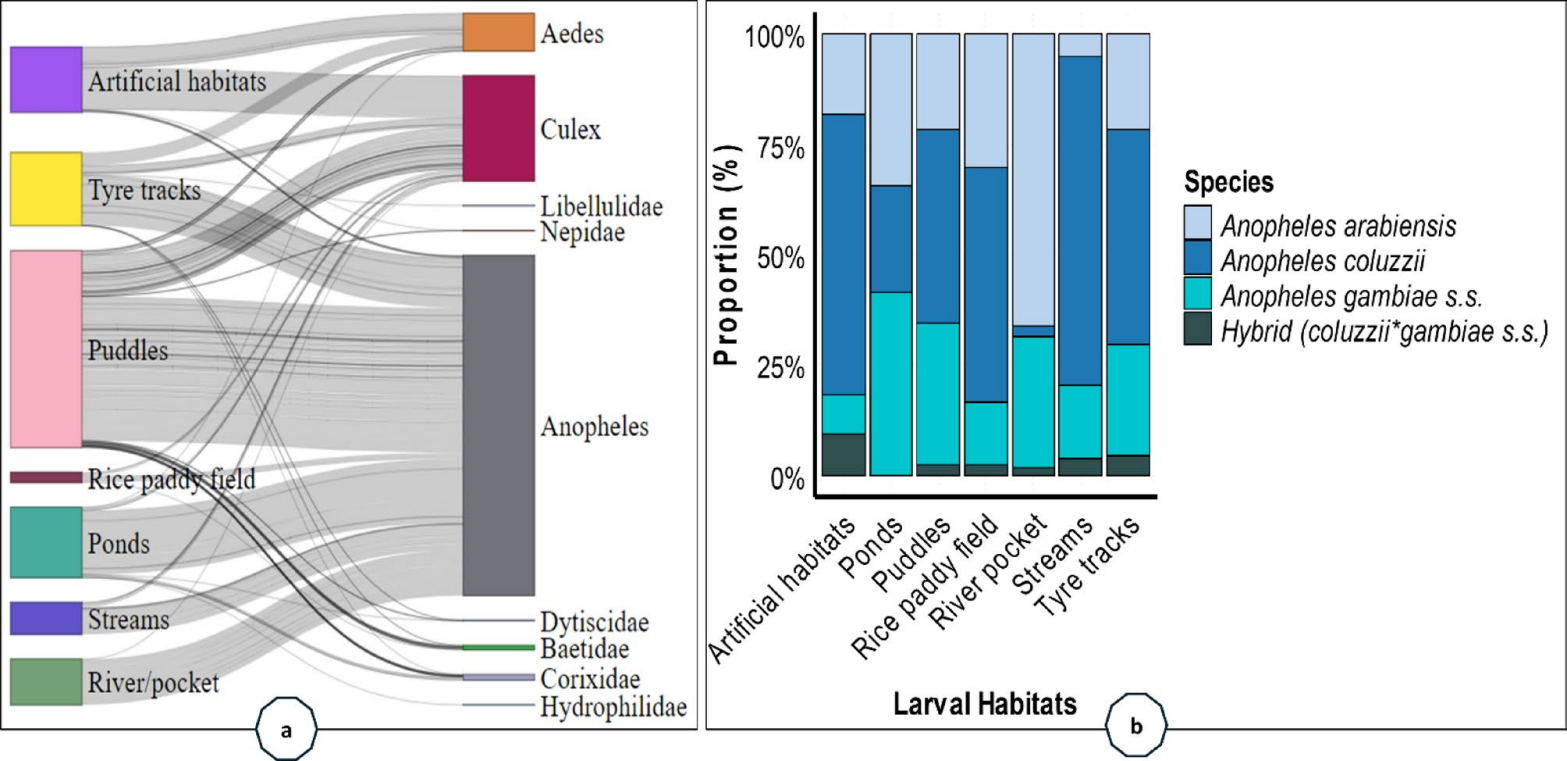
(**a**) Sankey plot of the association flows between larval habitats and species; (**b**) Proportions of the *Anopheles gambiae* species by larval habitat. Figure A provides the relationship between larval habitat and species, and their relative abundances and distributions, whereas Fig. 4b offers a more nuanced understanding of the community dynamics. For instance, the width of each flow indicates the strength of the association, meaning that wider flows suggest a greater abundance of a species in a particular habitat (**a**).

*Anopheles* spp. colonises puddles, ponds, tyre tracks, river/pocket, streams, and rice paddy fields rather than artificial habitats. Additionally, macroinvertebrates, such as Baetidae, Corixidae, Nepidae, Hydrophilidae, Dytiscidae and Libellulidae were present in the same larval habitats as *Anopheles* spp., even if they were present in low abundance (Fig. 2a). The proportional distribution of *An. gambiae* species within shared larval habitats (Fig. 2b) provides a better understanding of community dynamics. *Anopheles coluzzii*, *An. arabiensis*, *An. gambiae s.s*., and hybrid (*coluzzii × gambiae s.s*.) were found cohabiting at different proportions across almost all larval habitats.

### Ecological niche overlaps and species co-occurrence

**Fig. 3:**
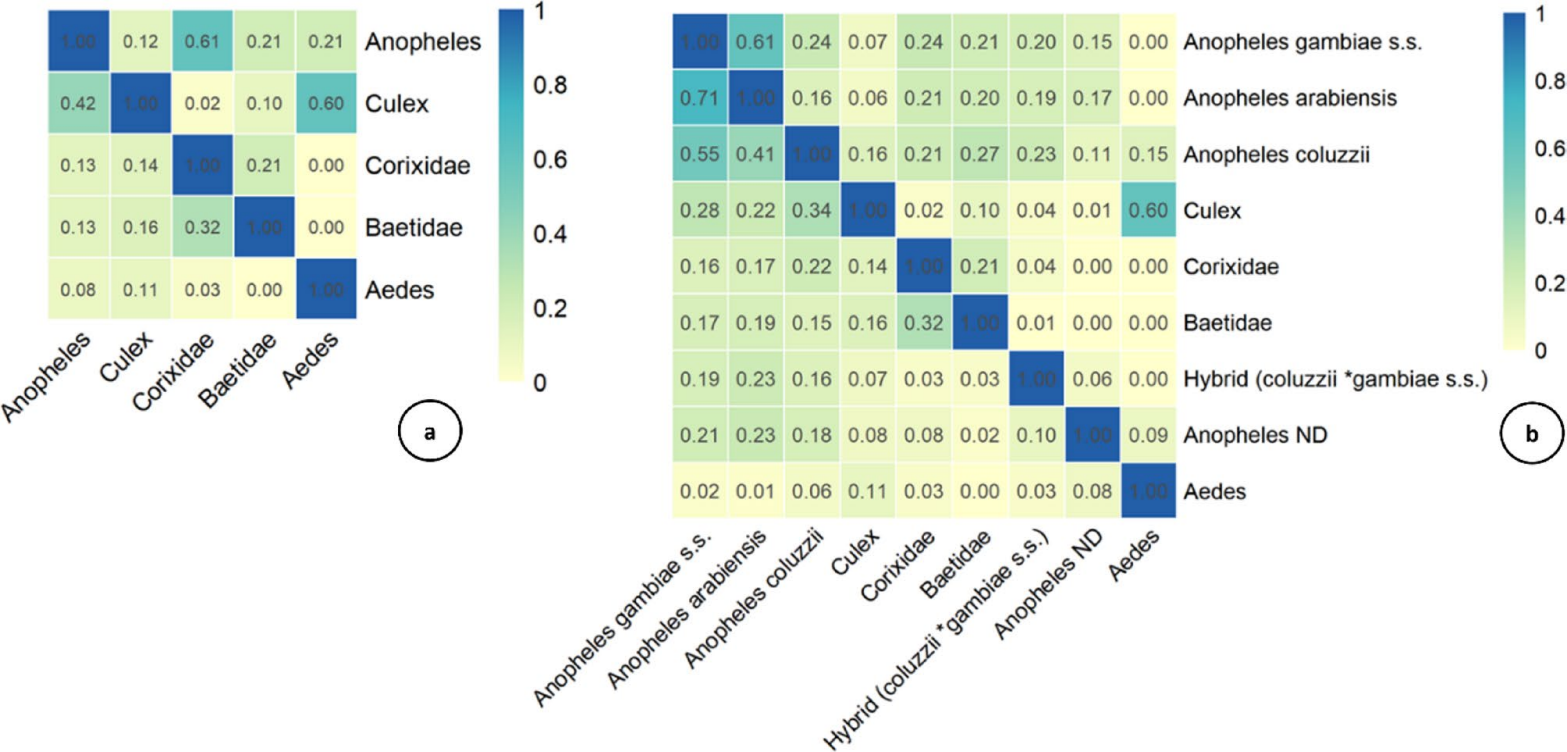
Combined heatmap of Pianka’s niche overlap index (upper triangle) and Jaccard’s co-occurrence index (lower triangle), (**a**) with *Anopheles* sp. identified morphologically and (**b**) with *Anopheles* species identified by PCR from a subsample. The colour scale illustrates the degree of niche overlap and spatial co-occurrence, with values ranging from very low (pale yellow) through intermediate levels (green to turquoise) to very high (deep blue) on a scale from 0 to 1.

The Pianka and Jaccard indices were calculated to assess co-occurrence patterns between *An. coluzzii*, other mosquito species, competitors, and predators sharing the same habitats (Fig. 3). Overall, most species pairs exhibited low to moderate Pianka values, while Jaccard values ranged from very low to high. *Anopheles gambiae s.s.* and *An. arabiensis* displayed low Pianka values alongside high and moderate Jaccard values, respectively, indicating niche differentiation and co-occurrence with *An. coluzzii*. Similarly, *Culex* spp. displayed low Pianka values and moderate Jaccard values, suggesting niche differentiation and partial co-occurrence. In contrast, species with low Pianka and Jaccard values, such as *Aedes* spp. and Baetidae (predators of *Anopheles* larvae), occupy distinct niches and exhibit minimal co-occurrence with *An. coluzzii* partially partitioned habitat. In addition to the pairwise analysis with *An. coluzzii*, a complementary assessment was performed using the genus *Anopheles*, i.e., before the identification of species by PCR, to provide a more extensive overview of co-occurrence and niche overlap patterns at the genus or family level. The results (Fig. 3a) reveal a high value of niche overlap but limited spatial co-occurrence between *Anopheles* spp. and Corixidae (predator species).

### Exposure assessment as a basis for hazard evaluation in the suppression of *Anopheles coluzzii*

An exposure score was calculated for each non-target species by combining Pianka and Jaccard indices with observed co-occurrence data. The score ranges from 0 (no exposure) to 1 (maximum exposure), providing a quantitative measure of potential contact or overlap with *An. coluzzii* (Table 2). Exposure scores differed among non-target organisms, with three species showing moderate exposure levels, including *An. gambiae s.s*., *An. arabiensis*, and *Culex* spp. Conversely, species such as Corixidae and Baetidae had low exposure scores, indicating comparatively limited spatial overlap with *An. coluzzii*.

**Table 2:** Exposure scores of the most exposed non-target organisms.

Non-target organisms	Pianka index	Jaccard index	Observed co-occurrence	Exposure score	Exposure level
*Anopheles gambiae s.s.*	0.237	0.546	0.679	0.463	Moderate
*Anopheles arabiensis*	0.158	0.409	0.487	0.332	Moderate
*Culex* spp.	0.158	0.337	0.436	0.295	Moderate
Corixidae	0.213	0.218	0.218	0.216	Low
Baetidae	0.272	0.146	0.154	0.199	Low
Hybrid (*coluzzii* × gambiaes.s.)	0.225	0.159	0.167	0.188	Low
*Anopheles* ND	0.110	0.182	0.205	0.160	Low
*Aedes* spp.	0.147	0.056	0.064	0.095	Low

## Discussion

This study aimed to evaluate ecological exposure and potential hazard to non-target organisms related to the suppression of *An. coluzzii*, through the combined analysis of niche overlap and co-occurrence patterns. Additionally, correlations between physicochemical parameters and taxa density were examined to understand how environmental factors may influence these interactions. Overall, the indices reveal a variety of co-occurrence patterns between *An. coluzzii* and NTOs, ranging from frequent coexistence despite niche differentiation to segregation across habitats. Exposure scores varied among them, with NTOs showing only moderate and low levels of exposure. These findings offer valuable insights into the ecological dynamics of mosquito populations, enhancing understanding of larval competition and identifying NTOs most likely to be affected by the suppression of *An. coluzzii*, which could serve as indicators for post-release monitoring.

The combined analysis of Pianka and Jaccard indices revealed varying patterns of niche overlap and spatial co-occurrence among *An. coluzzii* and sympatric taxa. Low Pianka values coupled with moderate Jaccard values, as observed between *An. coluzzii* and its two sibling species, but higher with *An. gambiae s.s.* than with *An. arabiensis*. These results indicate niche differentiation, associated with stable coexistence without intense competition, supporting an earlier study in Burkina Faso that showed a non-random distribution among these three sibling species^17,26^. *Anopheles coluzzii* was mainly associated with permanent and extensive larval habitats, such as rice fields. Furthermore, *An. gambiae s.s.* and *An. arabiensis* were mainly linked to temporary larval habitats created by rainfall. Therefore, studies have shown that *An. arabiensis* and *An. coluzzii* sometimes occupy similar larval habitats^27^. In this case, *An. arabiensis* significantly outcompetes *An. coluzzii* in mixed larval species, suggesting that even closely related mosquito species may often be more ecologically distinct than previously thought^27,28^. Conversely, low Pianka and moderate Jaccard values between *An. coluzzii* and *Culex* spp indicate neutral coexistence, and very limited or negligible direct competition. Onen et al.^29^, in their study in Uganda, reported partial co-existence between *Culex* spp. and *An. gambiae s.l.*, which each occupy distinct niches with no clear evidence of competition. Furthermore, Djimde et al. (2022) found that *An. coluzzii* cannot survive in breeding sites favourable to *Culex* spp^30^. This suggests that *An. coluzzii* and *Culex* spp. may sometimes coexist, but large-scale ecological replacement is unlikely, since their reproductive requirements overlap only partially and are not fully interchangeable.

Direct interactions between Corixidae and *An. coluzzii* were not detectable in our dataset. However, considering the wider *An. gambiae s.l.*, the combination of moderately high Pianka and low Jaccard values indicates shared habitat use with limited co-occurrence, suggesting spatial segregation likely driven by predation or behavioural avoidance. In fact, a study has shown that *Anopheles* larvae tend to reduce their activity and position themselves near walls or shallow edges, areas less accessible to predators such as Notonectidae, Dytiscidae, and Libellulidae, reinforcing the avoidance hypothesis^31^. *Anopheles gambiae s.s.* prefers temporary habitats that are generally low in predators, while *An. coluzzii* exploits refuges such as vegetation or floating debris in permanent habitats^26^. These adaptations limit direct interactions, which may explain the observed segregation. In addition to behavioural avoidance, effective predation may have reduced our exposure score, since larvae are either consumed or egg-laying females may avoid microhabitats occupied by predators. This pattern is consistent with predation-driven spatial segregation and highlights the potential role of aquatic predators in shaping mosquito larval distributions^29,32^. Therefore, if a predator needed to be monitored after suppression of *An. gambiae s.l.* through gene drive, Corixidae would be the most suitable candidate based on our results.

Following the niche overlap and co-occurrence index analyses, we examined the physicochemical characteristics of larval habitats to better understand the environmental factors influencing species distribution. This investigation provided valuable, insightful explanations for these patterns. Indeed, these factors can serve as reliable indicators of habitat preferences linked to key environmental conditions. *Anopheles coluzzii* exhibited a positive association with temperature and turbidity, as observed in a study in Cameroon, highlighting its ability to adapt to environments with varying pollution levels, especially in highly urbanised areas^33^. Conversely, turbidity was found to negatively affect the presence of *An. gambiae s.s.*^34^. Mosquito larvae are similar to fish like *Gambusia affinis* in their vulnerability to predation by visual predators in their aquatic habitats^35^. Therefore, high turbidity can hinder predators’ ability to detect larvae, increasing their survival chances^36^. This may explain why *An. coluzzii*, unlike *An. gambiae s.s.*, shows complete indifference to the presence of predators when choosing egg-laying sites^37^, indicating that the species is well adapted to such environments. Marubini et al. (2025) observed that *An. arabiensis* prefers to breed in moderately turbid water, unlike *An. coluzzii*, but it can also develop in clear water^38^. The results differ from other studies that linked *An. arabiensis* and *An. gambiae s.s.* with highly turbid waters^39^. Since food particles contribute to turbidity, these results can be suitable for mosquito larvae. In our study, electrical conductivity (EC) was positively associated with *An. arabiensis* in their larval habitats, despite the weak correlation. Studies conducted in Zambia by Musonda et al. (2019) and Nigeria by Akeju et al. (2022) showed a positive correlation between electrical conductivity and the abundance of *An. gambiae* and *An. funestus* larvae, indicating that moderate levels of EC may favour their survival while limiting adverse environmental factors^40,41^. However, other research offers a more nuanced view. For example, Chirebvu & Chimbari (2015) linked *An. arabiensis* and *An. gambiae s.s.* with habitats characterised by low EC^42^. In contrast, our observation of a positive, though weak, correlation between EC and *An. arabiensis* may reflect variability in the sampling sites. These findings suggest that there is an optimal range of electrical conductivity that enables *An. arabiensis* to thrive. EC levels that are neither too low nor too high could reduce competition or predation risk while remaining tolerable for this species.

In this study, we introduce a novel exposure score to estimate the potential ecological impact of *An. coluzzii* suppression on non-target organisms. The scores highlight differences in exposure among taxa, with *An. gambiae s.s.* showing the highest value, indicating a moderate level of exposure, followed by *An. arabiensis*, and *Culex* spp, while taxa such as Corixidae, Baetidae, and Hybrids (*An. coluzzii × An. gambiae s.s.*) exhibit low exposure levels. These patterns align with previously observed co-occurrence and niche overlap metrics, suggesting that species sharing similar habitats or resources with *An. coluzzii* are more likely to experience indirect effects. The high exposure score for *An. gambiae s.s.* could be linked to its close evolutionary and ecological relationship with *An. coluzzii*, from which it diverged relatively recently^43,44^. Although the hybrid form (*An. coluzzii × An. gambiae s.s.*) shows a low exposure score, this finding should be interpreted cautiously, especially in the context of gene drive strategies. Occasional hybridisation and gene flow between the two taxa, previously documented^45^, suggests that the spread of the introduced genetic construct beyond the initially targeted species is likely. Its functionality in other species remains to be confirmed, as the gene drive project will probably target the entire *An. gambiae* complex instead of only *An. coluzzii*.

Shifts in community structure have been observed following the suppression of dominant vector species in other systems. Decades ago, insecticide control led to the complete disappearance of *An. funestus* in southern Kenya and northern Tanzania, leading to an increase in *An. rivulorum* and *An. parensis* through reduced larval competition^18–20^. Similarly, in western Kenya, there was a significant decline in *An. gambiae s.s.*, which permitted a competitive release of *An. arabiensis*, allowing it to dominate^46^. These cases emphasise the risk that suppression of dominant species can create vacant ecological niches and enable colonisation by secondary vectors. In line with these dynamics, a previous study has shown that larval nutritional stress can have a carry-over effect on adult mosquito life history traits, including survival and fecundity^47^. These changes can also modify malaria transmission in complex and sometimes antagonistic ways, depending on mosquito density. By contrast, recent evidence further contextualises the role of predation. Metagenomic analyses from Ghana^48^ showed that *Anopheles* larvae broadly share resources with other filter-feeding taxa, whereas aquatic predators do not rely exclusively on them. This indicates that shifts in resource-based competition, rather than strong predator-prey dependence, are important.

Instead of predicting specific ecological outcomes, the proposed exposure score is designed to identify and prioritise non-target species for ecological monitoring after release. The exposure scoring system remains provisional and requires validation across different ecological contexts. While the study provides a practical framework to guide post-release ecological monitoring, some limitations should be acknowledged. The combined use of the indices can provide informative patterns and may suggest the relative strength of potential interactions between species. However, these inferences remain indirect and descriptive, and experimental or longitudinal studies are sometimes needed to confirm the nature and direction of underlying biotic interactions. Although physicochemical parameters were included to account for environmental filtering, the approach does not fully capture the complexity of aquatic community dynamics, including temporal variation.

Future research could expand this analysis by incorporating larger datasets and additional modelling techniques, such as null models for co-occurrence patterns^49^ or ecological niche factor analysis (ENFA), to improve understanding of interaction dynamics and environmental influences. Moreover, genomic surveillance tools such as ANOSPP amplicon panel or the use of eDNA combined with metabarcoding, offer promising prospects for detailing the full aquatic community^50–52^. Besides identifying macroinvertebrate species, these approaches may also uncover other biological interactions, such as microbial communities or predator-prey relationships, which could influence mosquito ecology^53^.

## Conclusion

Overall, this study provides an integrative and operational framework for assessing ecological exposure and potential hazard to non-target organisms following the suppression of *An. coluzzii*. By combining niche overlap and co-occurrence metrics, it identified organisms with varying levels of ecological connection, indicating interactions such as competition, spatial segregation, or predation. Various species, including *An. gambiae s.s.*, *An. arabiensis*, and *Culex* spp show moderate exposure scores, emphasising their potential as indicators for post-release environmental surveillance. The existence of hybrid forms (*An. coluzzii* x *An. gambiae s.s.*), although associated with low exposure scores, suggests possible gene flow, which, in a scenario aiming to target the *gambiae* complex, could facilitate the spread of suppression constructs across closely related species. Predatory organisms like Corixidae showed stronger associations at the level of the *An. gambiae s.l.* complex rather than individual subspecies, but it would still be interesting to consider them as ecological indicators, if such a complex were targeted, particularly since our exposure score tends to be lower for predators than competitors. This framework offers a valuable basis for predicting ecological interactions, guiding monitoring efforts, and informing risk assessments in vector control strategies.

## Methods

### Sampling sites

**Fig. 4:**
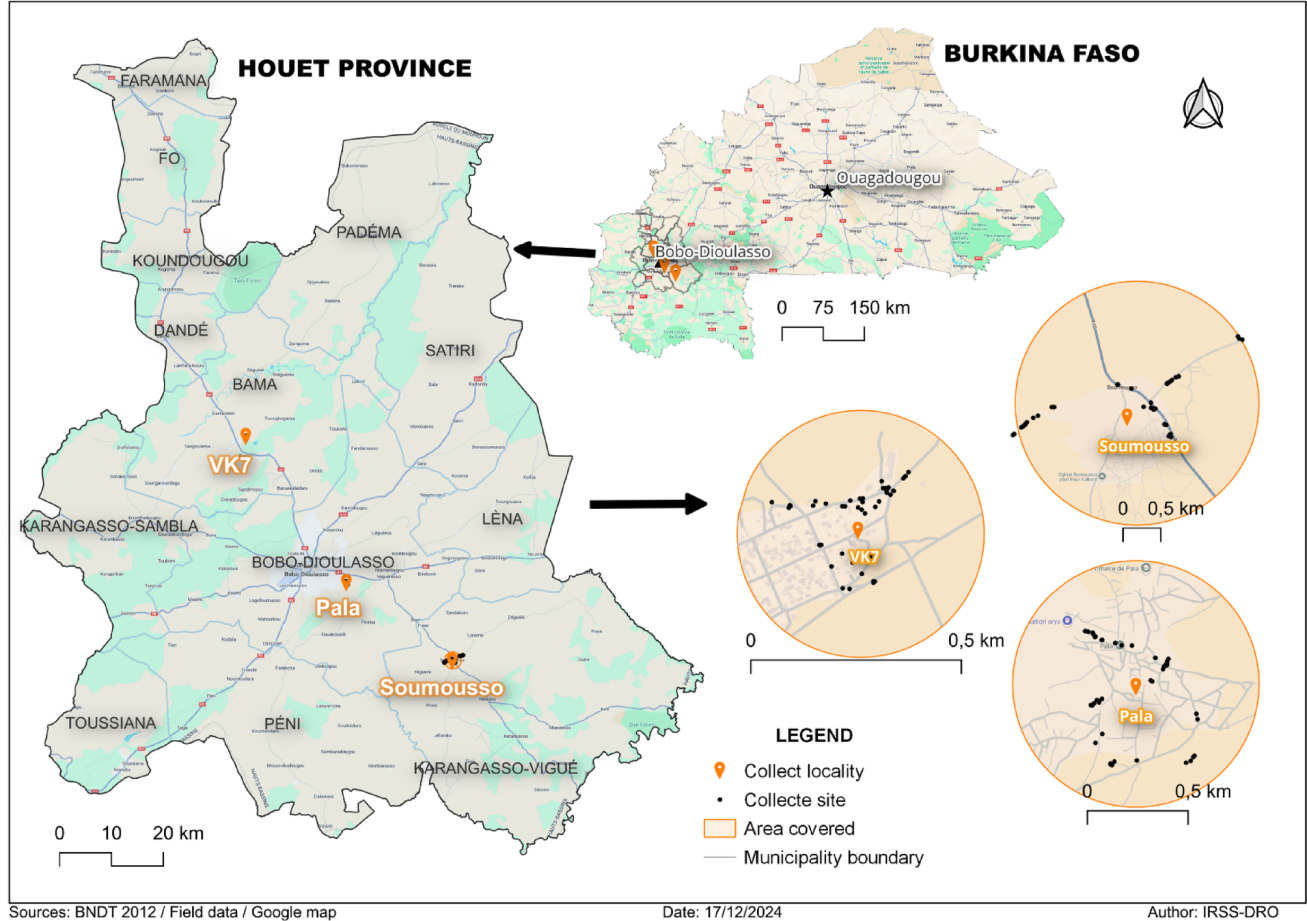
Map of the study area showing sample collection points.

Sampling was conducted at three sites: Bama/VK7 (11.406926°N−4.41175167°E), Soumousso (11.014304°N−4.048567°E), and Pala (11.150416°N−4.234323°E), in the western part of Burkina Faso (Fig. 4). These locations were chosen to represent the environmental, climatic, and socio-ecological diversity of the humid savannah surrounding Bobo-Dioulasso city. This region is especially significant for studying vector ecology and the patterns of vector-borne disease transmission. VK7 is within the municipality of Bama and has an estimated population of 31,215 inhabitants. It is part of an extensive irrigation scheme for rice cultivation, where perennial water flows year-round, supporting large aquatic systems conducive to mosquito breeding. Soumousso is a rural commune with approximately 7,669 residents, situated in a transitional agroecological zone featuring rainforests and semi-savannahs with open forests, which support a rich biodiversity. Pala, with about 2180 inhabitants, is a peri-urban area in Arrondissement 5 of Bobo-Dioulasso. This zone is prone to rapid, largely unplanned urban expansion characterised by a diverse interface between fragmented natural systems and growing human settlements. All three sites are within the Sudanese climatic zone, characterised by a tropical savannah climate with two distinct seasons: a rainy season from May to October, with an average annual rainfall exceeding 1,000 mm, and a dry season from November to April^54^. The dominant vegetation includes trees and open savannah forests, with gallery forests and wetlands along streams such as the Kou River, creating a mosaic of varied aquatic and semi-aquatic habitats. According to INSD (2023)^54^, agricultural activities- mainly cotton and similar crop cultivation- along with irrigation-based rice production in Bama and rain-fed agriculture in Soumousso, greatly affect local land-use patterns and the availability of mosquito breeding sites.

### Characterisation of mosquito breeding habitats

**Fig. 5oxy_comment_end:**
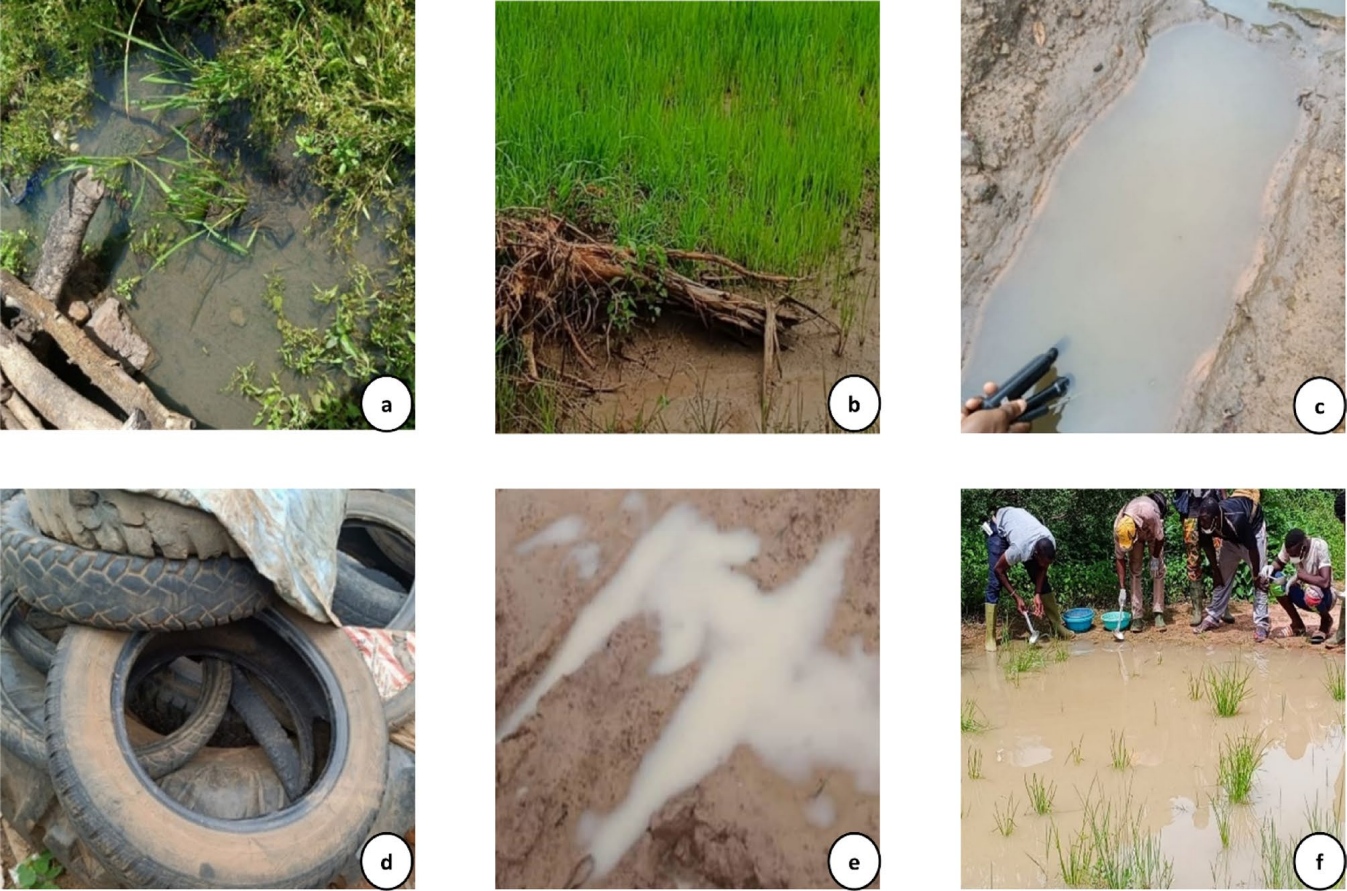
Larval habitats. Location from which samples were collected after assessing water physicochemical parameters ((**a**) stream; (**b**) rice paddy field; (**c**) puddle; (**d**) tyre; (**e**) tyre track; (**f**) pond).

Field surveys were carried out in Bama (Vk7), Soumousso, and Pala from July to October 2024 to identify and gather data on 138 mosquito larval habitats. To ensure spatial representativeness within each village, their areas were divided into four sections using a cross-shaped grid, with the main pathway acting as a central axis. In each section, breeding sites were systematically searched. This approach ensured a geographically representative sampling of breeding sites, reflecting their distribution across the different quadrants of each village. It revealed that they were primarily located along pathways and streams^55^. Mosquito larvae and other macroinvertebrates were collected from their natural breeding sites (Fig. 5), primarily using the dipping method. It revealed that they were mainly found along pathways and streams. Mosquito larvae and other macroinvertebrates were gathered from their natural breeding sites (Fig. 5), mainly using the dipping method. Sampling sites were categorised following approaches used in previous larval surveys^56^ and operationally adapted to local field conditions, allowing proportional adjustment of sampling effort. This approach is consistent with WHO/WHOPES guidance recommending that larval sampling effort be adapted to habitat type and size^57^. Based on this approach, larval habitats were classified into the following sizes:

- Small habitats (< 1 m in length): 2 squints (arm fully extended, bring sieve to the edge, lift content, and spread on the plate).
- Medium habitats (1–5 m): 4 squints from 2 positions on the edge of the larval habitat.
- Large habitats (> 5 m): 6 squints from 3 positions on the edge of the larval habitat.

The collected larvae and macroinvertebrates were brought to the laboratory for species identification. Before this, the small and large stages were separated, along with the macroinvertebrates, to prevent predation. Additionally, the small *Anopheles s.l.* larval stages were kept until L3 and L4 for identification. The mosquito larvae were identified using the morphological identification keys described by Robert et al. (2022)^58^. The macroinvertebrates were similarly identified using the morphological keys detailed by Gerber and Gabriel^55^. After identification, both the mosquito larvae and macroinvertebrates were stored in 80% alcohol for subsequent analyses. Molecular analyses were performed to identify the species within the *An. gambiae* complex, using the SINE 200X protocol^59^. During the mosquito larval survey, geographical coordinates, physicochemical parameters, and characteristics of each breeding site were also recorded. The physicochemical parameters, such as oxygen levels, conductivity, temperature, pH, and turbidity, were measured directly in the field before larval collection using a multiparameter handheld instrument (Lovibond SensoDirect 150). Subsequently, a water sample was taken from each deposit to measure turbidity (NTU) using the Turb^®^ 430 IR turbidimeter.

### Statistical analysis

All statistical analyses were performed using RStudio version 4.3.2 (RStudio, Inc., Boston, MA, USA, 2016). Averages and standard deviations of water physicochemical parameters (pH, turbidity, conductivity, % oxygen, and temperature) were calculated to highlight variations across larval habitats and observation periods. To compare means among multiple groups and evaluate their associations with larval habitat, the Kruskal-Wallis test was employed. The resulting p-values were computed and incorporated into summary tables using the summary package, enabling the assessment of the statistical significance of these differences. The non-parametric Spearman correlation was used to examine the relationship between mosquito species and physicochemical parameters, with data in supplementary file S2 and R code in supplementary file S3. Larval proportions for each genus and species were calculated as a percentage of the total larvae collected or used for molecular identification, and variations were analysed using Kruskal-Wallis tests. To assess the distribution of mosquito genera by site, a Fisher’s exact test with p-value simulation (10,000 replicates) was performed to explore the relationship between sampling sites and mosquito genera and species.

### Approach for non-target organisms risk assessment

Ecological risk assessment was carried out using an approach based on three complementary ecological metrics: exposure score that considers niche overlaps, spatial habitat similarity, and observed co-occurrence, each representing a dimension of habitat sharing and potential risk of interaction. This strategy has the advantage of assessing potential effects on NTOs after specific control of *An coluzzii*. Data (supplementary file S4) and R code (supplementary file S5) used to compute niche overlap, habitat similarity, co-occurrence rates, and the final exposure score for each non-target organism are available in the supplementary files.

*Pianka index* (Ojk), or Pianka’s niche overlap, is a symmetrical measure that indicates the level of similarity in resource category use between two species^60^. It is calculated by:

$$\:{O}_{jk}=\frac{\varSigma\:\left({P}_{ij}\cdot\:{p}_{ik}\right)}{\sqrt{\varSigma\:{p}_{ij}^{2}\cdot\:\varSigma\:{p}_{ik}^{2}}}$$

where:

- *O*_*jk*_: Niche overlap between species *j* and *k*.
- *P*_*ij*_ and *P*_*ik*_: Proportions of resource *i* used by species *j* and species *k*, respectively.

*Jaccard’s index* (J) was utilised to measure the similarity of species observed across different habitats to identify species co-occurrence^61^. It was calculated as follows:

$$\:J=\frac{a}{a+b+c}$$

- a: number of sites where both species are found.
- b: number of sites where the first species occurs and the second does not.
- c: number of sites where the second species is present and the first is absent.

*Observed co-occurrence* (OC = shared sites/target species sites) focuses on estimating the sites where the target species occurs, unlike the Jaccard index, which assesses the overall similarity. It answers when the target is present, and how often the non-target organism is also present. This measure is important to distinguish between general habitat sharing and the likelihood of direct interaction within the target’s specific habitat.

### Integrating metrics into a composite exposure score

Considering their relative ecological importance, weighting coefficients were assigned to compare each indicator against ecological exposure potential (Table 3). Niche overlap received the highest weight (40%), as it signifies a fundamental potential for ecological interaction through shared resource use and functional similarity. Habitat similarity, which accounts for spatial overlap as well as environmental opportunity for exposure^62,63^, and observed co-occurrence, reflecting actual interactions, albeit subject to environmental heterogeneity and sampling bias^64^, were both weighted 30%.

**Table 3:** Relative weighting of ecological indicators contributing to the composite exposure score.

Metric	Standard	Description	Question it answers
Niche overlap (Ojk)	40%	Similarity in resource use between species	Do they use the environment in the same way?
Habitat similarity (J)	30%	Proportion of habitats shared between two species	Do they live in the same types of places?
Observed co-occurrence (OC)	30%	Empirical frequency of species detected together within the same sampling units, reflecting realised spatial and temporal overlap	How often are two species found together?

Exposition score = (Ojk × 0.4) + (J × 0.3) + (OC × 0.3)

To aid in interpreting exposure scores, values were classified into four levels according to their relative magnitude (Table 4). This classification does not suggest direct ecological effects but offers a standardised scale for comparing potential exposure across taxa.

**Table 4:** Classification scale for exposure score interpretation.

Score range	Exposure level	Ecological interpretation	Potential impact
0.75–1.00	Very high	Strong ecological and spatial similarity	Likely major impact
0.50–0.75	High	Significant niche overlap	Moderate to high impact
0.25–0.50	Moderate	Partial interaction or occasional overlap	Limited impact
0.00–0.25	Low	Ecological segregation	Negligible impact

## Supplementary Information

Below is the link to the electronic supplementary material.

Supplementary Material 1

Supplementary Material 2

Supplementary Material 3

Supplementary Material 4

Supplementary Material 5

Supplementary Material 6

## Data Availability

Data are provided within the manuscript or supplementary information files S2 and S4. R Scripts or codes for correlation analyses and exposure score calculation are provided in supplementary files S3 and S5.
