# Supplementary material for: Ecological analysis of mosquito larval communities in Burkina Faso to inform environmental monitoring of genetic control programs
Source: Sci Rep. 2026 Jan 13;16:5091. doi: 10.1038/s41598-026-35602-6 (PMC12877188; doi:10.1038/s41598-026-35602-6)
Supplement: Supplementary file 1 — Supplementary Material 1 [file 41598_2026_35602_MOESM1_ESM.docx]

**Supplementary_file_S1:** Raw Spearman correlation outputs, including full correlation matrices (species x species and environmental x species), corresponding p-values, and significance levels used in the analyses.

**Supplementary_file_S2:** Complete raw dataset collected during the study, including all environmental, entomological, and ecological variables used for statistical analyses.

**Supplementary_file_S3:** R script used to perform the Spearman correlation analyses based on the complete raw dataset.

**Supplementary_file_S4:** Processed dataset used to calculate the exposure score presented in the manuscript.

**Supplementary_file_S5:** Exposure-score, Pianka index, Jaccard index, and observed co-occurrence R script.
